# Lenvatinib inhibits intrahepatic cholangiocarcinoma via Gadd45a-mediated cell cycle arrest

**DOI:** 10.1007/s12672-023-00631-4

**Published:** 2023-02-23

**Authors:** Xia Yan, Dan Wang, Zhouyu Ning, Zhi-qiang Meng

**Affiliations:** 1 Department of Oncology, Shanghai Cancer Center, Fudan University, 270 Dong An Road, Shanghai, 200032 China; 2 Present Address: Department of Cancer Center, Shanxi Bethune Hospital, Third Hospital of Shanxi Medical University, Taiyuan, Shanxi China

**Keywords:** Lenvatinib, Intrahepatic cholangiocarcinoma, Cell cycle arrest, Gadd45a, Patient-derived xenografts

## Abstract

**Purpose:**

To evaluate the anticancer activities of lenvatinib in ICC and its possible molecular mechanisms.

**Methods:**

Patients-derived xenograft (PDX) model and cell line-derived xenograft (CDX) model were both used for the in vivo study. For in vivo work, ICC cell lines were applied to analyze the effect of Lenvatinib on cell proliferation, cell cycle progression, apoptosis, and the molecular mechanism.

**Results:**

In the present study, we found that lenvatinib dramatically hindered in vivo tumor growth in ICC patient-derived xenograft models. In addition, by using in vitro experiments in ICC cell lines, we found that lenvatinib dose- and time-dependently inhibited the proliferation of ICC cells and induced cell cycle arrest in the G_0_/G_1_ phase. Transcriptional profiling analysis further applied indicated that lenvatinib might inhibit cell proliferation through the induction of cell-cycle arrestment via activating of Gadd45a, it was evidenced by that the knockout of Gadd45a significantly attenuated the cycle arrest induced by lenvatinib, as well as the inhibitory effect of lenvatinib on ICC.

**Conclusion:**

Our work first found that lenvatinib exerted an excellent antitumor effect on ICC, mainly via inducing Gadd45a-mediated cell cycle arrest. Our work provides evidence and a rationale for the future use of lenvatinib in the treatment of ICC.

**Supplementary Information:**

The online version contains supplementary material available at 10.1007/s12672-023-00631-4.

## Introduction

Intrahepatic cholangiocarcinoma (ICC) is a malignant epithelial tumor of the liver that is uncommon. In the past decade, even though the outlook for most solid tumors has gotten better, the prevalence of ICC has been increasing [1, 2]. For ICC patients, surgery is still the best long-term survival option. However, most ICC patients have early nonspecific symptoms and therefore lose the chance to undergo surgical treatment because ICC has already developed into an advanced stage by the time of diagnosis. The standard treatment for ICC is chemotherapy based on platinum and gemcitabine, however, it provides only a modest survival benefit [3–7]. Therefore, new therapeutic targets and drugs are urgently required to enhance the prognosis of individuals with ICC.

Fibroblast growth factor receptor (FGFR), which belongs to the family of receptor tyrosine kinases, can be activated by ligand binding, leading to dimerization and autophosphorylation of the intracellular domains of the receptor and receptor-related ligands [8]. Recent studies have shown that FGFRs were overexpressed in most solid tumors, including gastric cancer, pancreatic cancer, oesophageal cancer, and liver cancer. Studies have found that the prognosis of patients with FGFR overexpression is usually poor [9–11]. In ICC, changes in FGFR have been identified in 6–50% of patients, and these changes are related to an improvement in survival [12–14]. In addition, it has been confirmed that ICC patients with FGFR mutations are biologically more active than those patients with wild-type FGFRs. [15].

As an oral multitarget tyrosine kinase inhibitor (TKI), lenvatinib could exert its antitumor effect by impacting FGFR1-4, VEGFR1-3, PDGFRα, KIT- and RET-related angiogenesis, cell proliferation, and apoptosis. it was initially approved by the Food and Drug Administration (FDA) and the European Medicines Agency (EMA) in 2015 for the treatment of invasive, locally advanced, or metastatic differentiated thyroid cancer, and subsequently for the treatment of advanced renal cell carcinoma and unresectable hepatocellular carcinoma [16, 17]. Recently, its clinical efficacy in ICC has been increasingly noticed. As a pan-FGFR inhibitor, lenvatinib is expected to give ICC patients a new chance and has a better therapeutic effect by extending progression-free survival and overall survival times [18, 19]. But the effectiveness of lenvatinib and how it might work at the molecular level have not yet been fully figured out.

Ours is the first study to assess the anticancer properties of lenvatinib in ICC and its potential molecular pathways. This study provides encouraging evidence and a rationale for the clinical therapeutic application of lenvatinib in the treatment of patients with ICC.

## Material and methods

### Tumor xenografts in nude mice

The Ethics Committee on Animal Experiments of Fudan University approved the animal experiments, which were done in compliance with the “Regulations on the Administration of Laboratory Animal Affairs” (2017 edition).

#### Patient-derived xenograft (PDX) model

This study utilized human tumor tissue obtained from surgical patients with pathologically proven ICC. Ten- to twelve-week-old BALB/c-nu mice were purchased from Beijing Vital River Laboratory Animal Technology Co., Ltd. (Beijing, China). All the animals were kept in places with controlled temperatures, humidity, lighting (12 h of light, 12 h of dark), and easy access to food and water. Fresh tumor specimens measuring 2*2*2 mm were subcutaneously injected into each mouse's left flank. When the tumors reached a threshold volume, the tumor-bearing mice were killed, and the tumors were cut up and reinoculated into newborn mice according to the above method; these tumors were considered P1. Generally, tissues could be used for subsequent experiments after 2–3 passages, at which point they reached a stable state.

#### Cell line-derived xenograft (CDX) model

Six-week-old BALB/c-nu mice from Shanghai SLAC Laboratory Animal Co., Ltd., China, were housed in a way that kept them free of pathogens. 5*10^6^ ICC cells that were suspended in 50 μL PBS were mixed with 50 μL Matrigel and implanted subcutaneously into mice. At 14 days post-transplantation, tumor-bearing mice were randomized into four groups and treated with 3 mg/kg, 10 mg/kg, or 30 mg/kg with lenvatinib once daily for 28 days. The same volume of physiological saline was given to the mice in the control group.

In this study, when the body weight loss (BWL) of an individual mouse was 20%, that mouse was given a dosing break until its body weight returned to baseline (BWL, ≤ 10%). The length (a) and width (b) of tumor masses were measured with Vernier calipers twice a week, followed by the measurement of the mouse's body weight. Tumor volumes were calculated as V = 0.5 × a × b^2^. The tumor growth inhibition (TGI) rate was calculated as TGI (%) = [1−(Ti-T0)/(Vi-V0)] × 100, where Ti and Vi were the average tumor volumes of the experimental group and the control group after the end of drug program, and T0 and V0 were the average tumor volumes of the experimental group and the control group on the first day of administration. Similarly, the body weight (BW) of all tumor-bearing mice was measured twice a week. The ratio of body weight change (RCBW) after administration (BWi) was found by dividing the daily body weight by the mouse’s weight on day 0 (BW0): RCBW (%) = (BWi−BW0)/BW0 × 100. The nude mice were sacrificed through cervical dislocation the day after the last administration.

Following the Canadian Council on Animal Care (CCAC) guideline, the tumor burden should not exceed 2000 mm^3^. Furthermore, when the body weight loss (BWL) of an individual mouse was 20%, that mouse was given a dosing holiday(s) until its body weight returned to baseline (BWL, 10%). The protocols were approved by the Ethics Committee of Fudan University's Ethics Committee on Animal Experiments.

### Cells and reagents

The human ICC cell lines HCCC-9810 and RBE were obtained from the Type Culture Collection of the Chinese Academy of Sciences, Shanghai Institute of Cell Biology, Chinese Academy of Sciences (Shanghai, China). 3D Biomedicine Science and Technology Co., Ltd. provided the ICC-4389 cells (Shanghai, China). HCCC-9810 and RBE cells were cultured in RPMI 1640 medium (Biosera, NUAILLE, France). All media were supplemented with 10% FBS (Biosera, NUAILLE, France), and cells were grown in an incubator containing 5% CO2 at 37 degrees Celsius. The acquisition of Lenvatinib from Selleck (Houston, TX, USA).

### Cell viability assay

Using Cell Counting Kit-8 (CCK-8, Dojindo Laboratories, Japan) in accordance with the manufacturer’s instructions, cell viability and inhibition of cell growth was measured. The concentration of lenvatinib that resulted in a 50% reduction in cell viability was extrapolated using a nonlinear least squares curve that fitted the dose–response curves to produce 50% inhibitory concentration (IC50) values.

### Colony formation assay

In six-well plates, cells were first seeded and grown at a density of 1000 cells per well. After two weeks of cultivation, the cells were stained with 0.1% crystal violet (Sigma, St. Louis, United States) after being fixed with 4% paraformaldehyde. Using a light microscope, colonies with more than 50 cells were tallied.

### Flow cytometry

After harvesting and washing cells with PBS, 7% cold alcohol was added at 4 °C overnight. The cells were rinsed, suspended in PBS, and treated for 30 min at 4 °C with 100 mg/mL RNase and 40 mg/mL PI. On a FACS Calibur flow cytometer, the cell cycle distribution was analyzed (BD Bioscience, San Diego, CA, USA).

### Western blot analysis

Following treatment, cells were washed and lysed on ice in protease inhibitor-containing RIPA buffer. Using a bicinchoninic acid kit, protein concentrations were measured. Subsequently, 12 g of protein samples were loaded and separated using 10% SDS-PAGE to detect different proteins. The proteins were then transferred to a polyvinylidene fluoride membrane, which was then incubated with diluted primary antibodies (Abcam Inc., Cambridge, UK), including rabbit antibodies against Actin (1:5000, Proteintech, 23660-1-AP), CDK1 (1:1000, Cell Signaling Technology, 9116), CDK2 (1:1000, Cell Signaling Technology, 18048), CyclinB1 (1:1000, Proteintech, 55004-1-AP), Cyclin E (1:1000, Proteintech, 11554-1-AP), p21 (1:5000, Abcam Inc., ab109520) and Gadd45a (1:1000, Abcam Inc., ab180768), overnight at 4 °C. The blot was then washed thrice with PBS. The membrane was then treated for 1 h at 37 °C with a goat anti-rabbit immunoglobulin G (IgG) secondary antibody (HAF008, 1:5000; Novus Biologicals, LLC, Littleton, CO, USA) The proteins were finally observed using enhanced chemiluminescence (WBKLS0500; Merck KGaA). Using ImageJ software, semi-quantification was performed on blots (version no. k 1.45; National Institutes of Health).

### RNA preparation and quantitative real-time PCR

RNA was isolated from samples of cells and tumors. The TaKaRa PrimeScript RT reagent kit was utilized for reverse transcription (TaKaRa, Shanghai, China). Using an ABI 7900HT Real-Time PCR equipment, the expression of candidate genes was measured (Applied Biosystems, Inc., USA). The primer used in this study for Gadd45a was 5ʹ-AGAAGAGAGAGCATTCAATTCCA-3ʹ.

### RNA sequencing

Using the TRIzol reagent, total RNA was extracted from ICC cells treated with 0 M or 25 M lenvatinib (Invitrogen, CA, USA). Both cell lines were examined three times.

The sequencing of RNA was performed using the Illumina HiSeq 4000 sequencing machine. After data collection and preprocessing, the fragments per kilobase of exon per million mapped reads (FPKM) for each gene were analyzed.

### Plasmid and siRNA transfection

Following the manufacturer’s instructions, plasmid and siRNA were transfected using Lipofectamine 3000 Transfection Reagent (Thermo Fisher, MA, USA). The negative control consisted of an empty vector or a nonspecific siRNA. The sequence of the siRNA specific for Gadd45a (siRNA1) used in this study was 5ʹ-GCCGAAAGGGUUAAUCAUA-3ʹ. Samples were digested and used in other experiments 48 h after transfection.

### ICC tumor tissues and immunohistochemical (IHC) staining

Patients diagnosed with ICC at the Fudan University Shanghai Cancer Center (FUSCC, n = 90) were provided human ICC tumor tissue for histological examination. All operations were performed with approval from the Clinical Research Ethics Committee of the FUSCC, and each patient provided informed consent before any analyses. Two independent pathologists conducted strict pathological diagnoses and postoperative follow-ups. Animal tumor tissue sections were obtained from PDX/CDX model nude mice. Standard procedures were used to fix tumor samples with paraformaldehyde. The samples were then stained with antibodies against Ki67, Gadd45a, CyclinB1, and Cyclin E. The levels of protein expression were figured out by multiplying the number of positive (0, < 5% of total cells; 1, 5–25%; 2, 25–50%; and 3, > 50%) and intensity scores (0, no colouration; 1, pale yellow; 2, yellow; and 3, brown) and were classified as follows: negative (0, −); weakly positive (1–3, +); moderately positive (4–6, + +); and strongly positive (> 6, +  + +).

### Statistical analysis

We used SPSS 21.0 software from IBM Corp. in Armonk, New York, to look at the data. Experiments were done at least three times, and the data from the measurements are given as the mean plus the standard deviation. An unpaired t-test was used to compare data from two groups that followed a normal distribution or had the same amount of variation, while a paired t-test was used to compare data from ICC tissues and para-cancerous tissues. Data were compared among multiple groups via one-way analysis of variance (ANOVA) followed by Tukey’s post hoc test. Repeated-measures ANOVA followed by a Bonferroni post hoc test was utilized to compare data at different time points. P < 0.05 was considered to indicate a statistically significant difference.

## Results

### Lenvatinib displays potent antitumor efficacy in PDX models developed from ICC patient

**Fig. 1:**
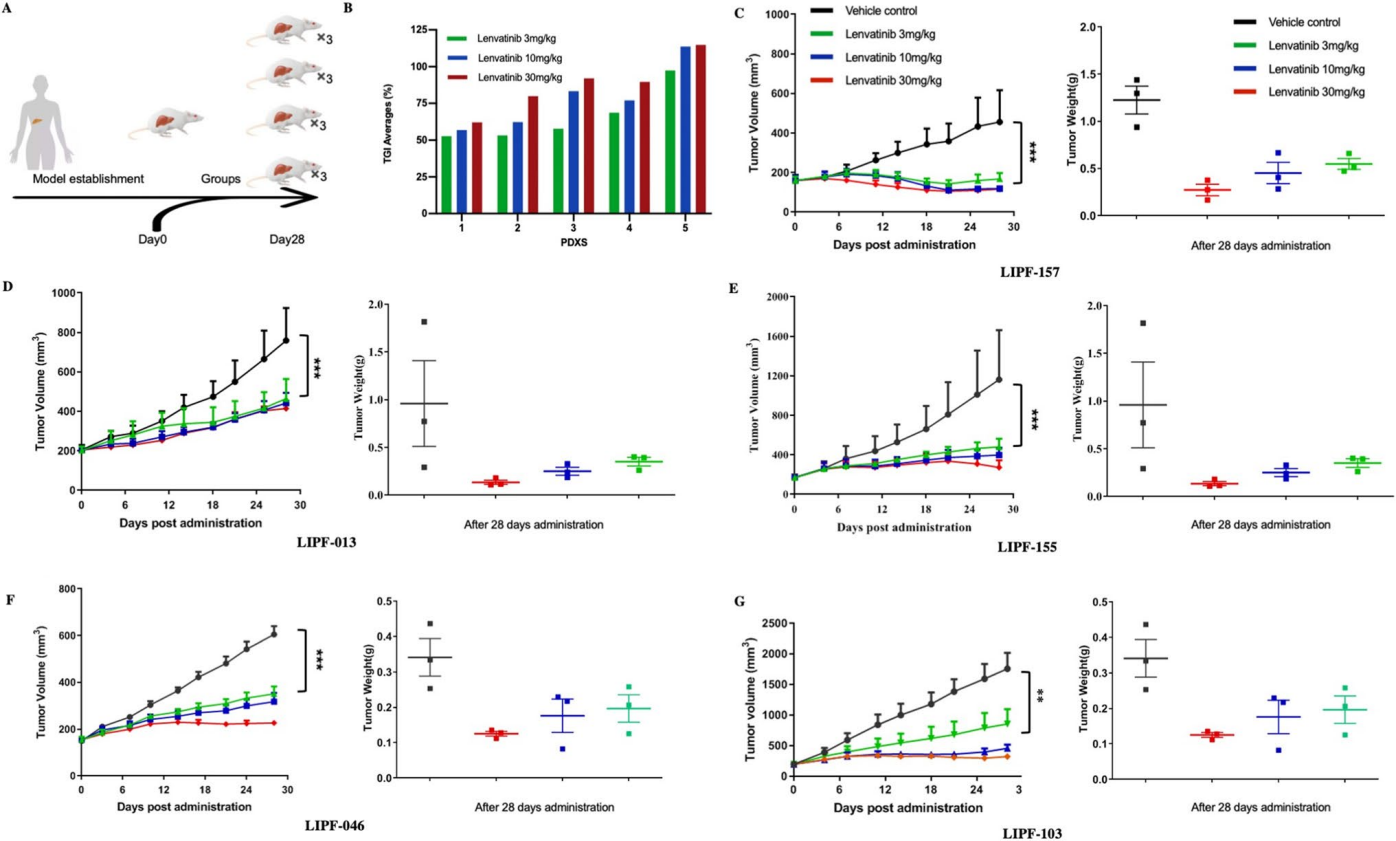
Lenvatinib exhibits effective antitumor activity in ICC PDX models. **a** Flow chart of in vivo experiments; **b** lenvatinib response after treatment in five PDX models; **c**–**g,** tumor growth curves and tumor weight box for each PDX case. *P < 0.05, **P < 0.01, ***P < 0.001, ****P < 0.0001

A PDX model is a tumor model created by transferring a patient's tumor tissue directly into immunodeficient mice. (Fig. 1a). This approach can not only retain the specific biological characteristics of the primary tumor but also results in good consistency with clinical research results. Thus, to preliminarily clarify the antitumor effect of lenvatinib in ICC, 5 PDX models were constructed. According to the model results, lenvatinib significantly inhibited the tumor growth of ICC PDXs, in which dramatic and significant increases in TGI were observed. However, the sensitivity of PDXs from different patients to lenvatinib varied (Fig. 1b). The weights of the tumors in the control group were also much higher than those in the lenvatinib group. (Fig. 1c-g). Compared with the control group, no significant difference in the relative change rate in body weight was noticed (Supplementary Fig. 1). There were also no significant changes with lenvatinib administration in diarrhea, appetite, or mental state in the PDX mouse model. Taken together, these results indicate that lenvatinib had effective antitumor activity against ICC and few side effects in vivo.

### Lenvatinib inhibits ICC cell growth in vitro

**Fig. 2:**
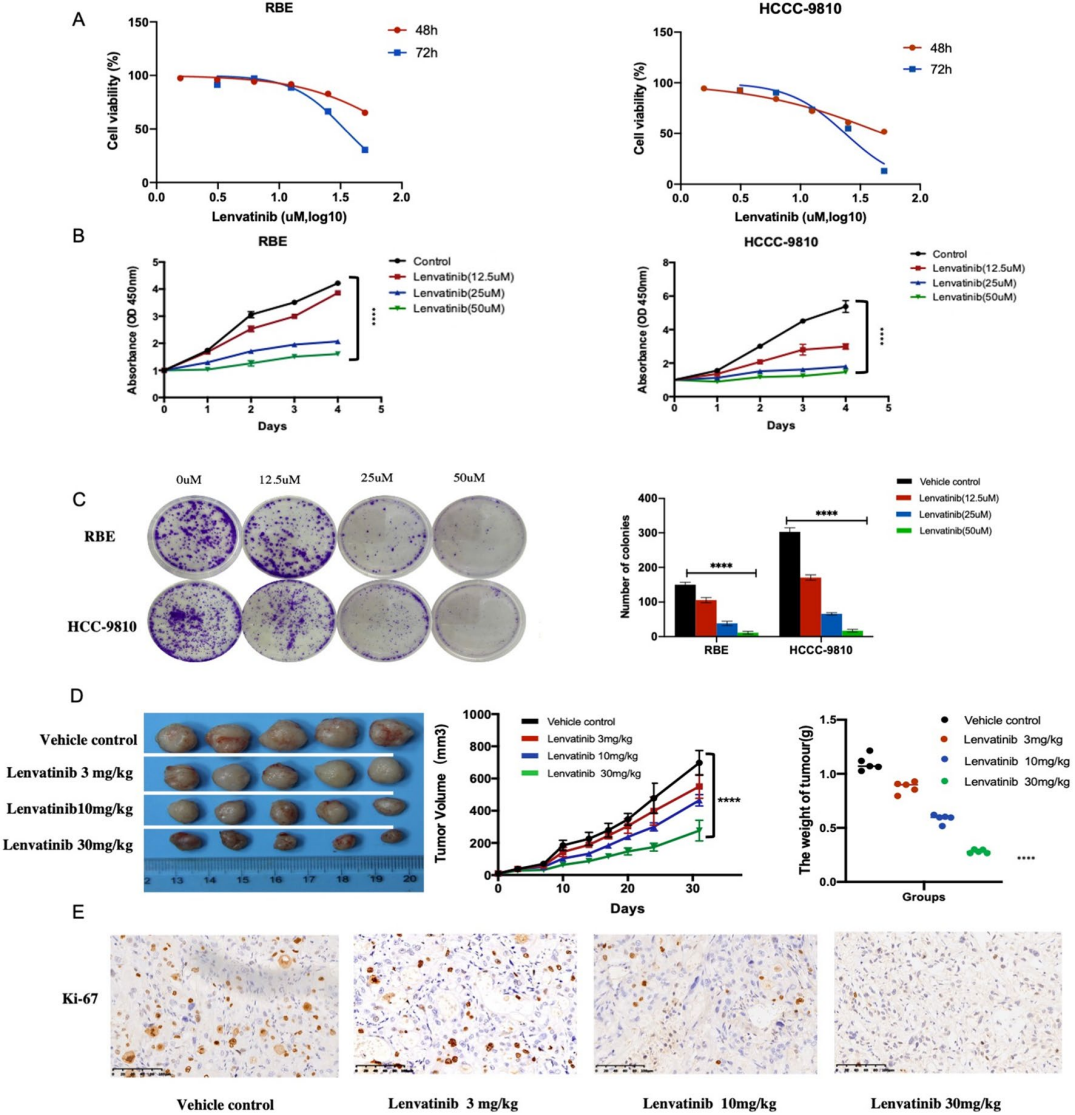
Lenvatinib inhibits ICC cell growth in vitro. **a** HCCC-9810 and RBE cells were treated with lenvatinib for 48 and 72 h. Cell viability was measured using CCK-8, and IC50 values were calculated. **b** CCK-8 assays were used to test the effects of lenvatinib on the viability of ICC cells. **c** Representative images of colony formation assays. **d** Tumor growth curves and tumor weights for the CDX model. **e** The expression of the proliferation marker Ki-67 was determined in tumor tissue sections from the xenografts using IHC (scale bar, 20 μm). *P < 0.05, **P < 0.01, ***P < 0.001, **** P < 0.0001

After we established that lenvatinib does have an inhibitory effect on ICC in vivo, we investigated the potential antitumor effect of lenvatinib using ICC cell lines (HCCC-9810 and RBE). Lenvatinib exerted anticancer effects in HCCC-9810 and RBE cells in a concentration-dependent manner, with decreased cell viability observed at high concentrations. The IC50 values of lenvatinib in HCCC-9810 and RBE cells were 22.63 μM and 34.09 μM at 72 h, respectively (Fig. 2a). Based on the observed IC50 values, lenvatinib was used at concentrations of 0 (vehicle control), 12.5, 25, and 50 µM in subsequent analyses. CCK-8 assay results showed that the rates of cell proliferation were inhibited after 48 h of lenvatinib treatment, and significant dose-dependent inhibitory effects between the concentration groups were detected in both cell lines after 72 h (Fig. 2b). Furthermore, the colony formation ability of the cell lines was significantly suppressed by treatment with increasing concentrations of lenvatinib, and the differences were significant (Fig. 2c; P < 0.05).

In addition, to examine whether lenvatinib can inhibit ICC cells in vivo, a subcutaneous CDX model was established. Consistent with the in vitro findings, mice treated with lenvatinib exhibited significant inhibition of tumor growth parameters, including tumor weight and tumor volume, compared to control mice. Furthermore, lenvatinib had no significant effect on mouse body weight (Fig. 2d). Moreover, staining for the proliferation marker Ki-67 in xenograft tumors collected 1 day after the last administration of lenvatinib was performed, and the number of Ki-67-positive cells in the lenvatinib-treated groups was reduced compared with that in the control groups (Fig. 2e).

### Lenvatinib induces cell cycle arrest in ICC

**Fig. 3:**
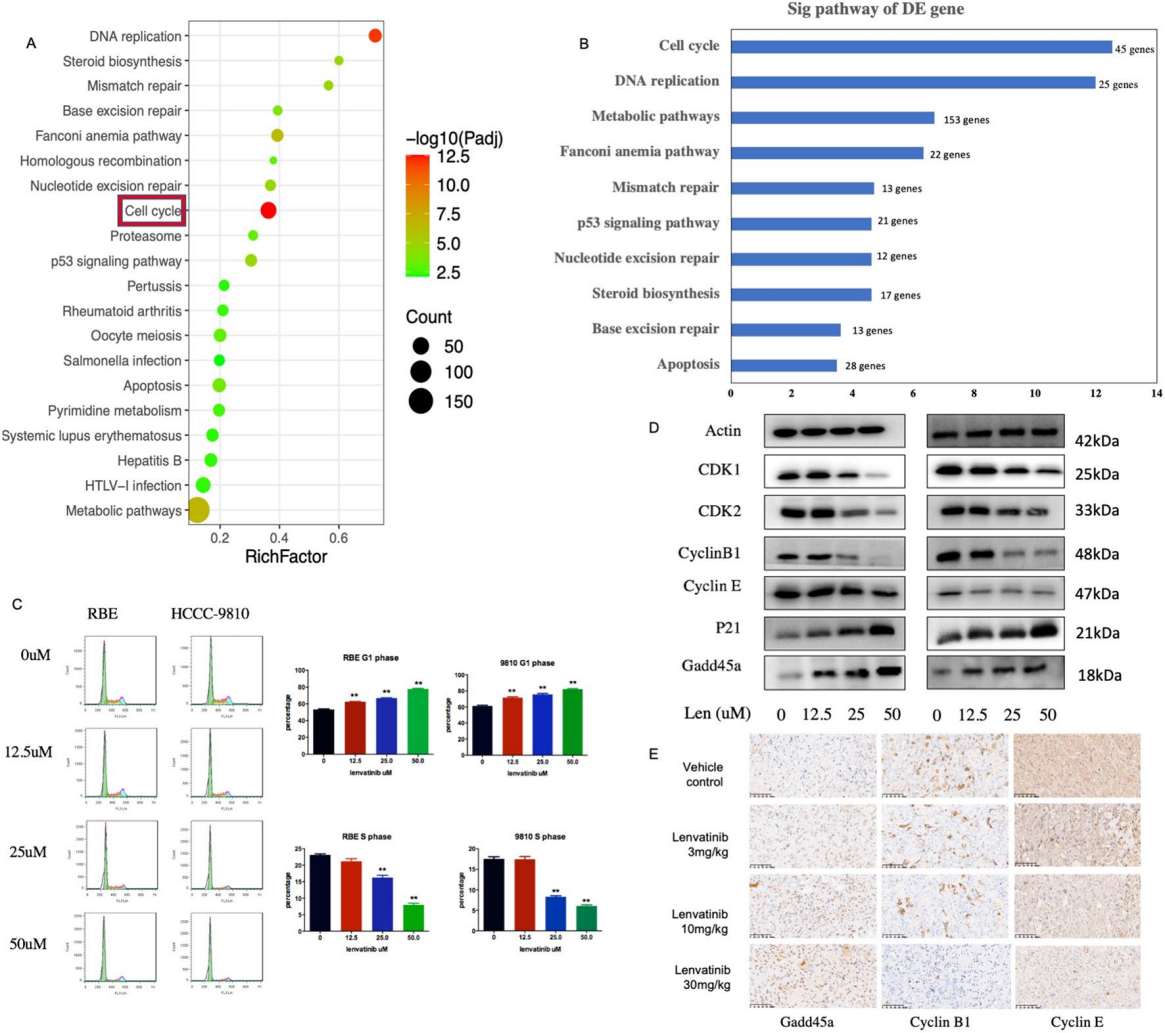
Lenvatinib induces cell cycle arrest in ICC. **a** and **b** KEGG analysis of differentially expressed genes after treatment with 25 μM lenvatinib (HCCC-9810); **c** Cells were treated with lenvatinib at the indicated concentration for 72 h and then stained with PI for flow cytometric analysis. Each experiment was performed in triplicate. **d** Expression of cell cycle-related proteins in ICC cell lines after treatment with a concentration gradient of lenvatinib for 72 h was detected using western blotting. **e** Cyclin B1 and Cyclin E expression were significantly downregulated and Gadd45a expression was upregulated after lenvatinib treatment, based on immunohistochemistry (IHC) analysis (scale bar, 20 μm). *P < 0.05, **P < 0.01, ***P < 0.001, **** P < 0.0001

RNA sequencing was performed to reveal the gene expression profiles of cells treated with lenvatinib or the control. Kyoto Encyclopedia of Genes and Genomes (KEGG) enrichment analysis based on significantly differentially expressed genes revealed a series of enriched signaling pathways, including cell cycle (Fig. 3a and b). Given that cell cycle progression is one of the essential factors for the proliferation of ICC cells, cells were then examined via flow cytometry after incubation with 0, 12.5, 25, or 50 μM lenvatinib for 72 h. Based on our results, dose‐dependent arrest in the G0/G1 phase of the cell cycle occurred in HCCC-9810 and RBE cells after treatment with lenvatinib. The G0/G1 phase cell percentages were 52.34%, 69.62%, 70.31%, and 77.23% (in RBE cells) and 63.34%, 75.43%, 78.4%, and 82.23% (in HCCC-9810 cells) at 0, 12.5, 25, and 50 μM lenvatinib, respectively (Fig. 3c). Moreover, these distributions were consistent with the observed levels of important proteins involved in the cell cycle, including CDK1/CyclinB1, CDK2/Cyclin E and p21/Gadd45a (Fig. 3d). IHC staining of tumor tissues indicated that the expression of CyclinB1 and Cyclin E was significantly suppressed in the group treated with lenvatinib (Fig. 3e), which was consistent with the in vitro findings. Collectively, our results indicate that lenvatinib can inhibit ICC cell proliferation by inducing cell cycle arrest.

### Lenvatinib upregulates Gadd45a in vitro and in vivo, and Gadd45a expression is positively correlated with ICC prognosis

**Fig. 4:**
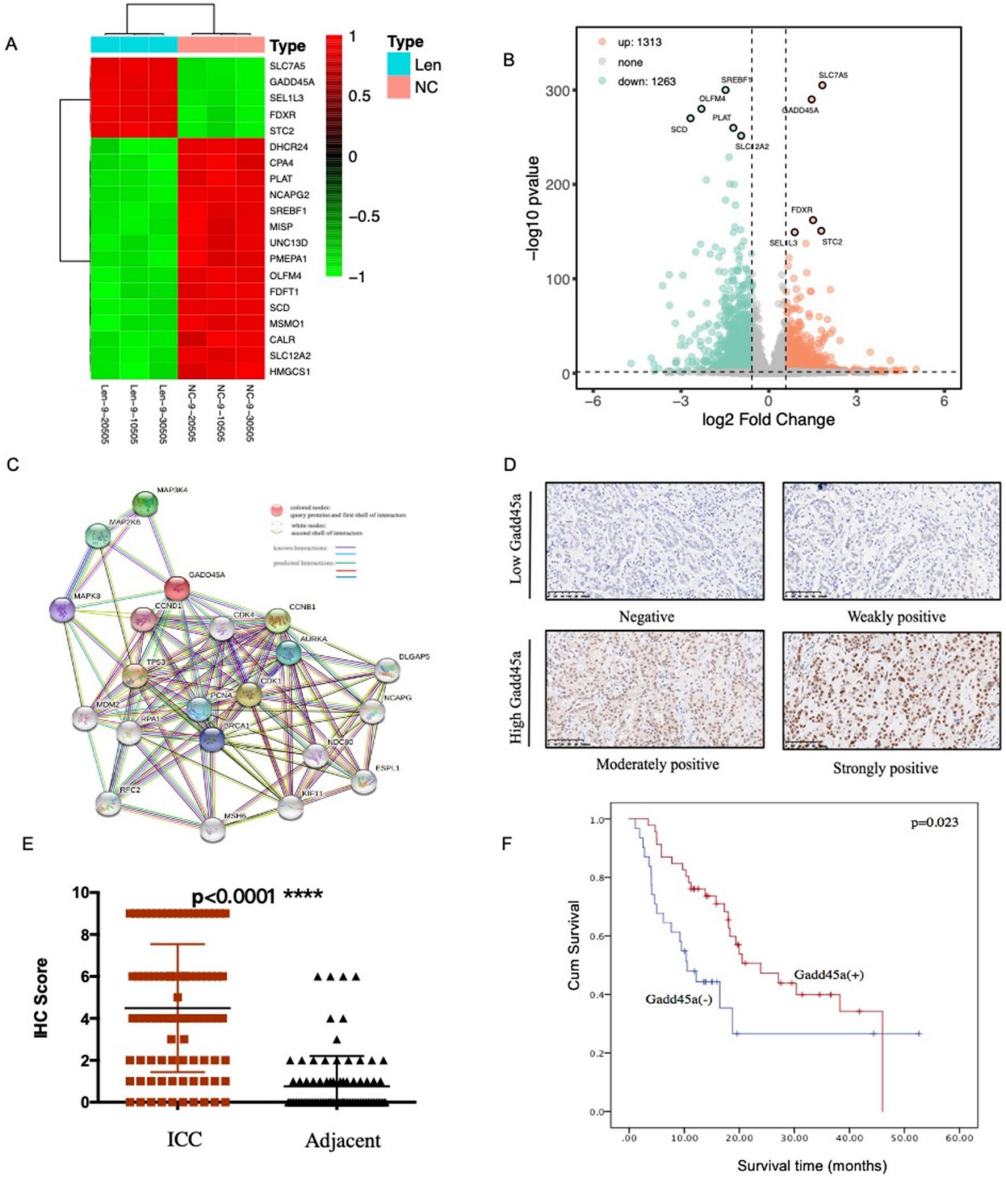
Lenvatinib upregulates Gadd45a in vitro and in vivo, and Gadd45a expression is positively correlated with ICC prognosis. **a** and **b** Heatmap and volcano showing clustering of the top differentially expressed genes (determined by RNA sequencing) between ICC cells treated with lenvatinib (25 μM) and control cells; **c **protein–protein interaction network of Gadd45a, based on String; **d** representative images of IHC staining for Gadd45a in ICC (scale bar, 20 μm); **e** Gadd45a expression in ICC and adjacent normal tissues, as determined by the IHC score (n = 90 pairs, ****P < 0.0001);** f** the OS of patients with ICC was assessed based on Gadd45a expression using Kaplan–Meier analysis (n = 77, *P = 0.023)

To further identify the key molecular targets of lenvatinib in ICC, clustering analysis was performed, and the results showed that the lenvatinib and control groups could clearly be distinguished according to the differentially expressed genes, with significant Gadd45a upregulation detected in the lenvatinib-treated groups (Fig. 4a–c). To preliminarily investigate the role of Gadd45a in ICC, tissue microarrays (TMAs) containing 90 pairs of patient samples were evaluated. According to the immunohistochemistry score, Gadd45a was expressed at higher levels in tumor tissues than in normal tissues (Fig. 4d and e). Moreover, after excluding 13 cases with incomplete clinical and follow-up data, the demographic and Clinical Characteristics of ICC patients were presented in Table 1. Kaplan–Meier survival curves revealed an obvious correlation between high Gadd45a expression and a better prognosis in ICC patients (p = 0.023; Fig. 4f). According to a Cox regression analysis, Gadd45a expression was an independent prognostic marker of ICC (Supplementary Table S).

**Table 1:** Demographic and clinical characteristics of patients

Variables	Gadd45a (−) (56)	Gadd45a ( +) (31)	*p*-value
Age (year)			
≤ 60	27	19	0.270
> 60	29	12	
Gender			
Female	26	13	0.822
Male	30	18	
T stage			
1	12	10	0.337
2	42	21	
3	2	0	
Lymph node status			
Negative	39	22	1.000
Positive	17	9	
TNM stage			
I	12	8	0.889
II	26	14	
III	18	9	
Tumor differentiation			
Well	10	2	0.088
Moderate	39	28	
Poor	7	1	

### Gadd45a is a key factor by which lenvatinib induces cell cycle arrest in ICC

**Fig. 5:**
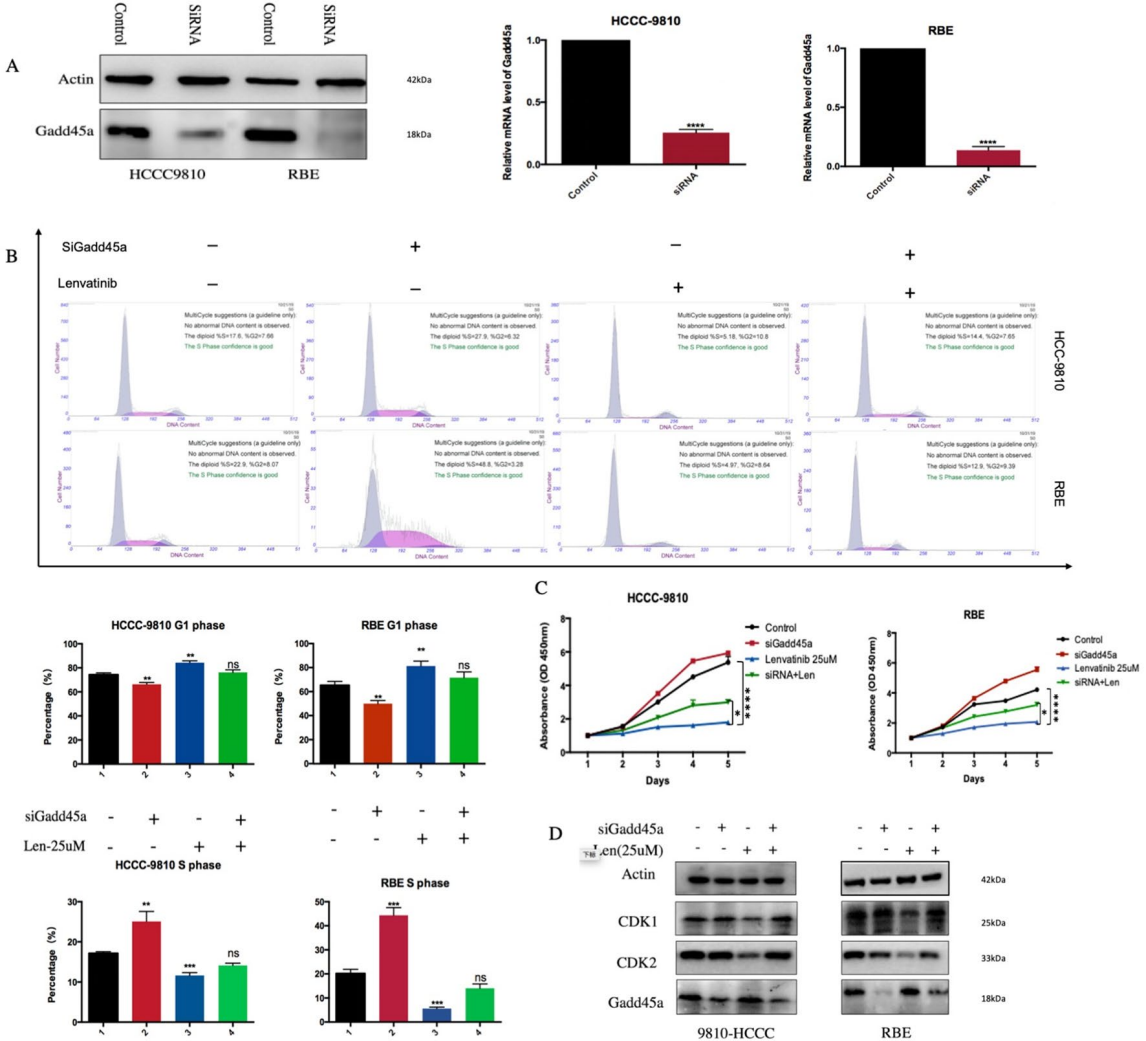
Gadd45a is a key factor by which lenvatinib induces cell cycle arrest in ICC. **a** Gadd45a protein and mRNA silencing in ICC cells was confirmed by immunoblotting analysis and quantitative RT-PCR, respectively; **b** and** c** the proportion of cells in each phase of the cell cycle and the viability of cells in Gadd45a-silenced and Gadd45a-scramble cell lines following lenvatinib treatment (all statistics are based on comparison with the control group); **d** immunoblotting analysis of Gadd45a, CDK1 and CDK2 levels in ICC cells with Gadd45a silencing or scramble following lenvatinib treatment. The grouping of blots cropped from different parts of the same gel or from different gels, fields, or exposures is divided with white space. *P < 0.05, **P < 0.01, ***P < 0.001, **** P < 0.0001

**Fig. 6:**
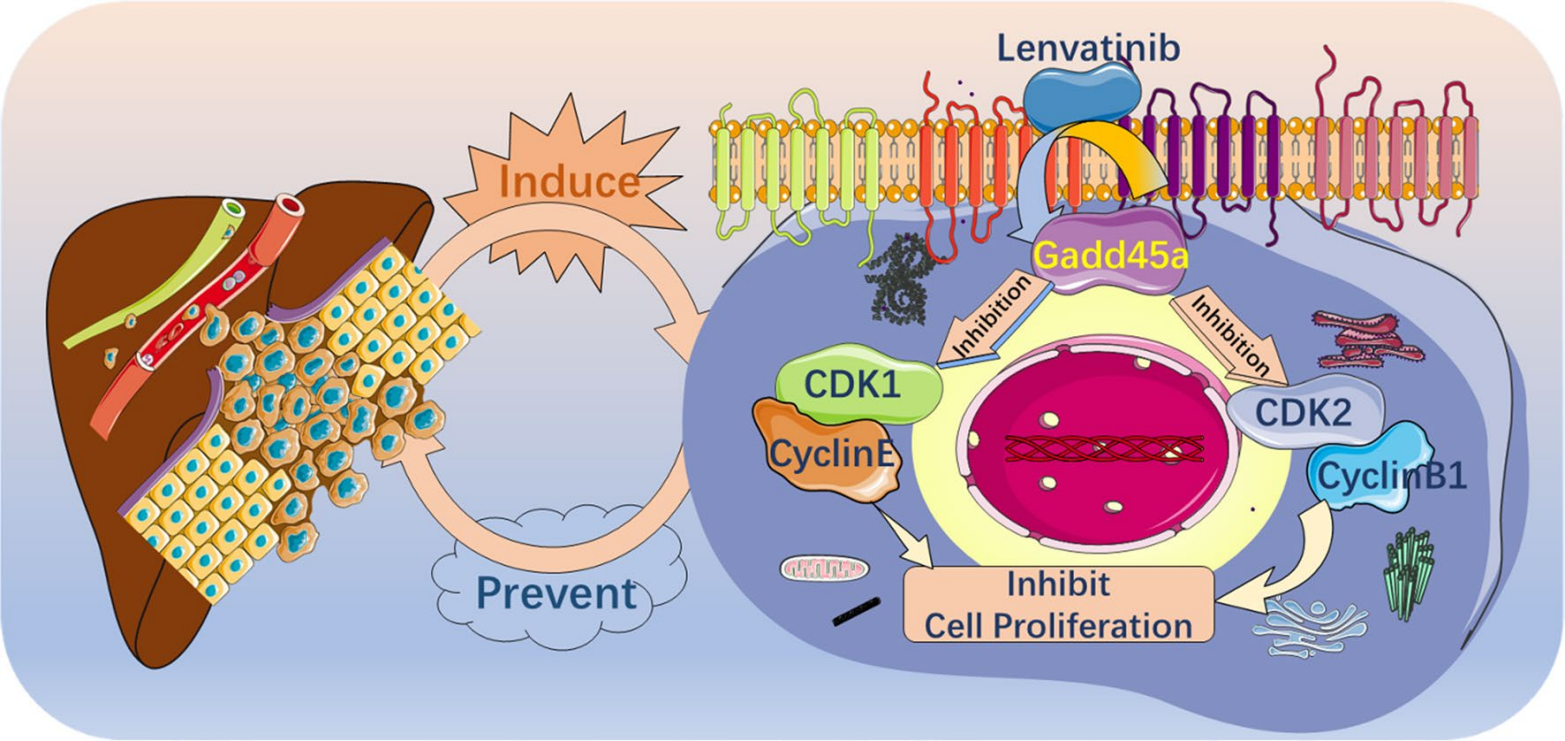
The possible mechanism of lenvatinib in ICC

Given the crucial findings described above, we hypothesized that activated Gadd45a is involved in the process of ICC inhibition by lenvatinib. To assess this possibility, HCCC-9810 and RBE cells were transfected with Gadd45a-specific siRNA, and the transfection efficiency was confirmed by western blotting and quantitative RT-PCR (Fig. 5a). As shown in Fig. 5b and c, Gadd45a silencing obviously weakened the inhibitory effects of lenvatinib on cell cycle progression and cell proliferation in both ICC cell lines. Compared with the control group, the gadd45a silencing group showed a significantly reduced proportion of G-phase cells, while the proportion of G1-phase cells was increased with lenvatinib treatment. However, no difference was found when lenvatinib was applied to gadd45a-silenced cells, suggesting that Gadd45a plays a crucial role in cell cycle regulation mediated by lenvatinib. Moreover, the observed levels of CDK1 and CDK2 in Gadd45a-silenced cells following lenvatinib treatment were consistent with the aforementioned results (Fig. 5d), indicating that lenvatinib inhibits ICC cell proliferation via Gadd45a-mediated cell cycle arrest (Fig. 6).

## Discussion

As an oral multitarget TKI, lenvatinib has been found to be effective against thyroid cancer, hepatocellular carcinoma, and renal cancer [20]. Recently, its efficacy in ICC has been increasingly noticed, but there is no relevant research, and the mechanism remains largely unclear. In this study, we preliminarily confirmed that (1) lenvatinib had excellent antitumor effects in ICC cell lines and ICC PDX models; (2) lenvatinib inhibited ICC cell proliferation through induction of cell cycle arrest; (3) Gadd45a, a DNA damage-inducing gene, was the key factor in the regulation of the cell cycle by lenvatinib; and (4) patients with high expression of Gadd45a had a better prognosis.

PDX models using patient tumors as the direct tissue source are valid preclinical models for oncology drug development and drug response prediction. Studies have found that the biological characteristics of the primary tumor can not only be preserved in a primary transplantation model but also be well preserved in a post-transplantation model. In PDX models of colon cancer, the genetic phenotypes and mutations of primary tumors were consistently and stably expressed in the subculture model. Efficacy experiments conducted with PDX models have as great as 90% consistency with the results of clinical efficacy studies [21, 22]. In our study, to determine the value of lenvatinib in ICC treatment, 5 PDX models of ICC were constructed, and the results showed that lenvatinib could significantly inhibit the growth of ICC compared with control treatment, and no significant difference in body weight was noted among the groups. This result suggests that the agent should be safe and effective for ICC patients in clinical practice.

As one of the important components of tumor cell proliferation, cell cycle progression has been demonstrated to play important roles in the occurrence and development of many tumors. Cyclin-dependent protein kinases (CDKs) and cyclin-dependent protein kinase inhibitors (CKIs) are known to be the key regulatory loci of the cell cycle, in which cyclins can positively regulate CDKs, while CKIs negatively regulate CDKs [23, 24]. In our work, dose‐dependent cell cycle arrest in G0/G1 phase was obvious in ICC cell lines after treatment with lenvatinib, which was consistent with the observed levels of important proteins involved in the cell cycle, including CDK1/CyclinB1 and CDK2/Cyclin E. Based on transcriptome analysis, we found that Gadd45a was significantly upregulated after treatment with lenvatinib. As a member of the growth arrest and DNA damage gene (Gadd) family, Gadd45a was previously reported to be closely related to the prognosis of tumor patients and was found to be a cell cycle regulator [25–27]. On the one hand, activated Gadd45a can directly inhibit the expression of cell cycle-related proteins (CDKs/CKIs) to prevent cell cycle progression; on the other hand, it can inhibit the phosphorylation of cyclins/CDKs by inducing the expression of p21 [28, 29]. In our present study, similar results were observed; increased Gadd45a expression was found to be correlated with better OS in ICC and might serve as a prognostic biomarker of ICC. In addition, loss of Gadd45a expression significantly attenuated the cell cycle arrest induced by lenvatinib, as well as the inhibitory effect of lenvatinib against ICC, indicating that Gadd45a is a key link of lenvatinib in ICC treatment. However, the potential upstream mechanism by which lenvatinib regulates Gadd45a was not fully elucidated here. As an important downstream molecule of p53 in cell cycle regulation, Gadd45a can be regulated in a p53-dependent manner, while the expression of p53 in tumor cells can be significantly inhibited by the PI3K/Akt signaling pathway [30–32]. Thus, given recent findings showing that activation of the PI3K/Akt signaling pathway is closely related to the binding of FGFRs to their ligands [33–35], we speculate that the regulation of Gadd45a by lenvatinib may rely on the upstream PI3K/Akt/p53 signaling pathway, which will be further investigated in our future work.

In summary, our PDX model-based investigations provided attractive preclinical results. Therefore, prospective clinical trials should be performed to evaluate the efficacy of lenvatinib treatment in patients with ICC. More data should be collected to verify the antitumor activity and side effects of lenvatinib before its clinical application in ICC patients.

## Conclusions

The results of our present study indicate that lenvatinib has excellent antitumor activity in ICC cells and can inhibit ICC proliferation through induction of Gadd45a-mediated cell cycle arrest.

## Supplementary Information

Supplementary file1 (TIF 150 KB)

Supplementary file2 (DOCX 15 KB)

## Data Availability

The datasets used and/or analyzed during the current study are available from the corresponding author upon reasonable request.

## References

[1] Bertuccio P, Malvezzi M, Carioli G, Hashim D, Boffetta P, El-Serag HB (2019). Global trends in mortality from intrahepatic and extrahepatic cholangiocarcinoma. J Hepatol.

[2] Zhang H, Yang T, Wu M, Shen F (2016). Intrahepatic cholangiocarcinoma: epidemiology, risk factors, diagnosis and surgical management. Cancer Lett.

[3] Le Roy B, Gelli M, Pittau G, Allard MA, Pereira B, Serji B (2018). Neoadjuvant chemotherapy for initially unresectable intrahepatic cholangiocarcinoma. Br J Surg.

[4] Massani M, Nistri C, Ruffolo C, Bonariol R, Pauletti B, Bonariol L (2015). Intrahepatic chemotherapy for unresectable cholangiocarcinoma: review of literature and personal experience. Updates Surg.

[5] Mavros MN, Economopoulos KP, Alexiou VG, Pawlik TM (2014). Treatment and prognosis for patients with intrahepatic cholangiocarcinoma: systematic review and meta-analysis. JAMA Surg.

[6] Rizvi S, Khan SA, Hallemeier CL, Kelley RK, Gores GJ (2018). Cholangiocarcinoma—evolving concepts and therapeutic strategies. Nat Rev Clin Oncol.

[7] Yuan L, Luo X, Lu X, Huang B, Cai Q (2016). Liver resection for intrahepatic cholangiocarcinoma in AJCC-stage IV: an evaluation of the survival benefit and prognostic accuracy of current AJCC staging system on N and M classification. Oncol Rep.

[8] Babina IS, Turner NC (2017). Advances and challenges in targeting FGFR signalling in cancer. Nat Rev Cancer.

[9] Goyal L, Saha SK, Liu LY, Siravegna G, Leshchiner I, Ahronian LG (2017). Polyclonal secondary FGFR2 mutations drive acquired resistance to FGFR inhibition in patients with FGFR2 fusion-positive cholangiocarcinoma. Cancer Discov.

[10] Grünewald S, Politz O, Bender S, Héroult M, Lustig K, Thuss U (2019). Rogaratinib: a potent and selective pan-FGFR inhibitor with broad antitumor activity in FGFR-overexpressing preclinical cancer models. Int J Cancer.

[11] Hegab AE, Ozaki M, Kameyama N, Gao J, Kagawa S, Yasuda H (2019). Effect of FGF/FGFR pathway blocking on lung adenocarcinoma and its cancer-associated fibroblasts. J Pathol.

[12] Starska K, Forma E, Lewy-Trenda I, Stasikowska-Kanicka O, Skóra M, Bryś M (2018). Fibroblast growth factor receptor 1 and 3 expression is associated with regulatory PI3K/AKT kinase activity, as well as invasion and prognosis, in human laryngeal cancer. Cell Oncol.

[13] Goyal L, Shi L, Liu LY, de la Fece Cruz F, Lennerz JK, Raghavan S (2019). TAS-120 overcomes resistance to ATP-competitive FGFR inhibitors in patients with FGFR2 fusion-positive intrahepatic cholangiocarcinoma. Cancer Discov.

[14] Lamberti D, Cristinziano G, Porru M, Leonetti C, Egan JB, Shi CX (2019). HSP90 inhibition drives degradation of FGFR2 fusion proteins: implications for treatment of cholangiocarcinoma. Hepatology.

[15] Rizvi S, Gores GJ (2017). Emerging molecular therapeutic targets for cholangiocarcinoma. J Hepatol.

[16] Kudo M, Finn RS, Qin S, Han KH, Ikeda K, Piscaglia F (2018). Lenvatinib versus sorafenib in first-line treatment of patients with unresectable hepatocellular carcinoma: a randomised phase 3 non-inferiority trial. Lancet.

[17] Makker V, Rasco D, Vogelzang NJ, Brose MS, Cohn AL, Mier J (2019). Lenvatinib plus pembrolizumab in patients with advanced endometrial cancer: an interim analysis of a multicentre, open-label, single-arm, phase 2 trial. Lancet Oncol.

[18] Qiu B, Chen T, Sun R, Liu Z, Zhang X, Li Z (2019). Sprouty4 correlates with favorable prognosis in perihilar cholangiocarcinoma by blocking the FGFR-ERK signaling pathway and arresting the cell cycle. EBioMedicine.

[19] Touat M, Ileana E, Postel-Vinay S, André F, Soria JC (2015). Targeting FGFR signaling in cancer. Clin Cancer Res.

[20] Taylor MH, Lee CH, Makker V, Rasco D, Dutcus CE, Wu J (2020). Phase IB/II trial of lenvatinib plus pembrolizumab in patients with advanced renal cell carcinoma, endometrial cancer, and other selected advanced solid tumors. J Clin Oncol.

[21] Byrne AT, Alférez DG, Amant F, Annibali D, Arribas J, Biankin AV (2017). Interrogating open issues in cancer precision medicine with patient-derived xenografts. Nat Rev Cancer.

[22] Hidalgo M, Amant F, Biankin AV, Budinská E, Byrne AT, Caldas C (2014). Patient-derived xenograft models: an emerging platform for translational cancer research. Cancer Discov.

[23] Engeland K (2018). Cell cycle arrest through indirect transcriptional repression by p53: I have a DREAM. Cell Death Differ.

[24] Pietenpol JA, Stewart ZA (2002). Cell cycle checkpoint signaling: cell cycle arrest versus apoptosis. Toxicology.

[25] Hildesheim J, Fornace AJ (2002). Gadd45a: an elusive yet attractive candidate gene in pancreatic cancer. Clin Cancer Res.

[26] Ishiguro H, Kimura M, Takahashi H, Tanaka T, Mizoguchi K, Takeyama H (2016). GADD45A expression is correlated with patient prognosis in esophageal cancer. Oncol Lett.

[27] Pietrasik S, Zajac G, Morawiec J, Soszynski M, Fila M, Blasiak J (2020). Interplay between BRCA1 and GADD45A and its potential for nucleotide excision repair in breast cancer pathogenesis. Int J Mol Sci.

[28] Han N, Yuan F, Xian P, Liu N, Liu J, Zhang H (2019). GADD45a mediated cell cycle inhibition is regulated by P53 in bladder cancer. Onco Targets Ther.

[29] Liu LQ, Tian FJ, Xiong Y, Zhao Y, Song JB (2018). Gadd45a gene silencing by RNAi promotes cell proliferation and inhibits apoptosis and senescence in skin squamous cell carcinoma through the p53 signaling pathway. J Cell Physiol.

[30] Abraham AG, O'Neill E (2014). PI3K/Akt-mediated regulation of p53 in cancer. Biochem Soc Trans.

[31] Fu G, Dai J, Li Z, Chen F, Liu L, Yi L (2020). The role of STAT3/p53 and PI3K-Akt-mTOR signaling pathway on DEHP-induced reproductive toxicity in pubertal male rat. Toxicol Appl Pharmacol.

[32] Wang X, Simpson ER, Brown KA (2015). p53: protection against tumor growth beyond effects on cell cycle and apoptosis. Cancer Res.

[33] Chen R, Li D, Zheng M, Chen B, Wei T, Wang Y (2020). FGFRL1 affects chemoresistance of small-cell lung cancer by modulating the PI3K/Akt pathway via ENO1. J Cell Mol Med.

[34] Okada T, Enkhjargal B, Travis ZD, Ocak U, Tang J, Suzuki H (2019). FGF-2 attenuates neuronal apoptosis via FGFR3/PI3k/Akt signaling pathway after subarachnoid hemorrhage. Mol Neurobiol.

[35] Packer LM, Geng X, Bonazzi VF, Ju RJ, Mahon CE, Cummings MC (2017). PI3K inhibitors synergize with FGFR inhibitors to enhance antitumor responses in FGFR2(mutant) endometrial cancers. Mol Cancer Ther.

