# Supplementary figures and images for: Lenvatinib inhibits intrahepatic cholangiocarcinoma via Gadd45a-mediated cell cycle arrest
Source: Discov Oncol. 2023 Feb 23;14:26. doi: 10.1007/s12672-023-00631-4 (PMC9950313; doi:10.1007/s12672-023-00631-4)

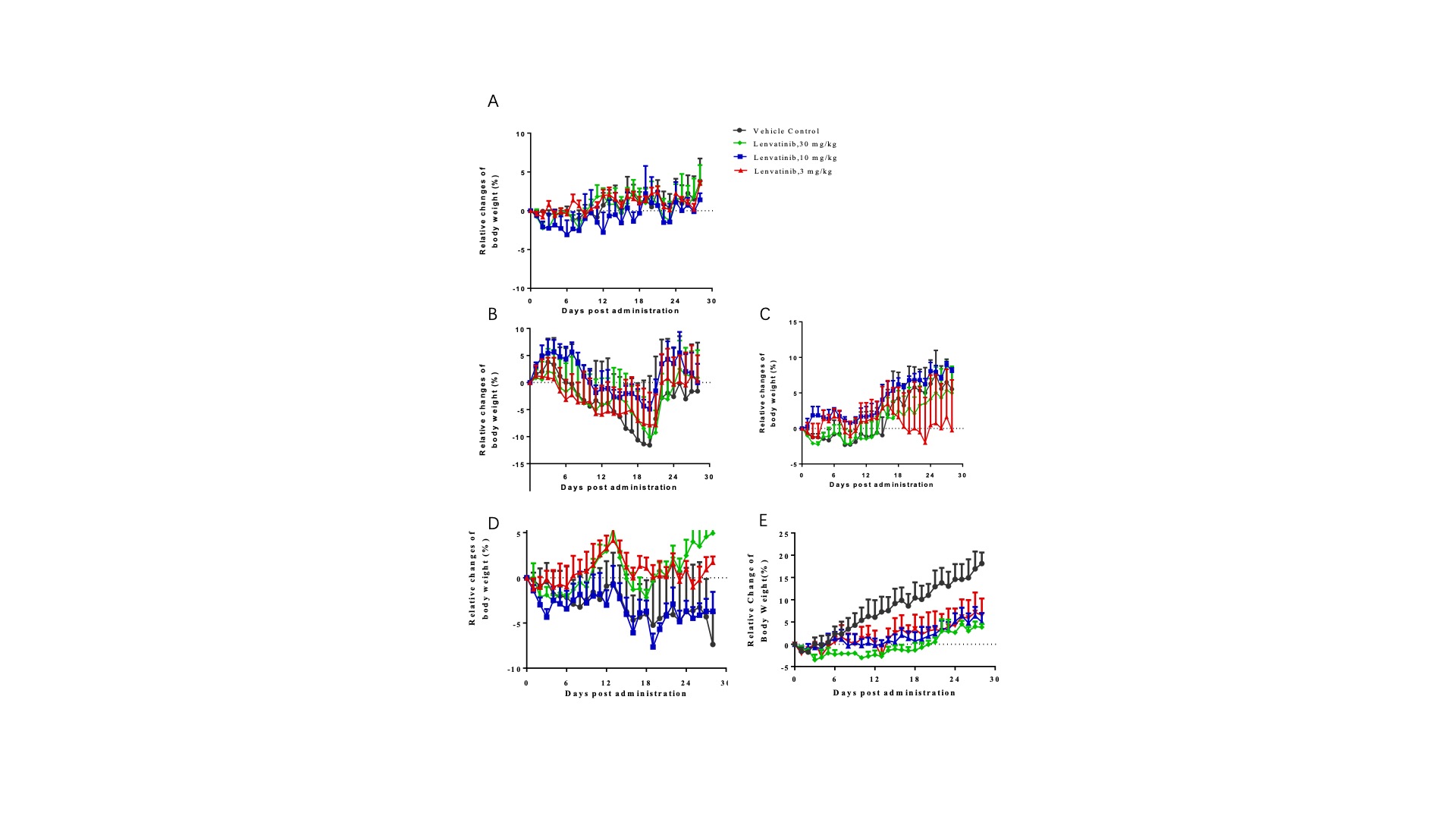

Supplement: Supplementary file 1 — Supplementary file1 (TIF 150 KB) [file 12672_2023_631_MOESM1_ESM.tif]
