# Supplementary material for: Lenvatinib inhibits intrahepatic cholangiocarcinoma via Gadd45a-mediated cell cycle arrest
Source: Discov Oncol. 2023 Feb 23;14:26. doi: 10.1007/s12672-023-00631-4 (PMC9950313; doi:10.1007/s12672-023-00631-4)
Supplement: Supplementary file 2 — Supplementary file2 (DOCX 15 KB) [file 12672_2023_631_MOESM2_ESM.docx]

| **Supplementary Table : Univariate and multivariate analysis for OS of ICC patients using the COX proportional hazards model** | | | | |
| --- | --- | --- | --- | --- |
| **Features** | **Univariate Analysis** | | **Multivariate Analysis** | |
|  | HR (95%CI) | P-value | HR (95%CI) | P-value |
| **Age (yr)** |  |  |  |  |
| ≤60 | 1 (/) |  |  |  |
| >60 | 0.933 (0.526-1.655) | 0.813 |  |  |
| **Gender** |  |  |  |  |
| Female | 1 (/) |  |  |  |
| Male | 1.030 (0.775-1.369) | 0.839 |  |  |
| **T stage** |  |  |  |  |
| 1 | 1 (/) |  | 1 (/) |  |
| 2 | 1.837 (0.942-3.583） | 0.074 | 1.550 (0.780-3.078) | 0.259 |
| 3 | 5.945 (1.289-27.41） | ***0.022*** | 1.642 (0.298-9.054) | 0.336 |
| **Lymph node status** |  |  |  |  |
| Negative | 1 (/) |  | 1 (/) |  |
| Positive | 2.375(1.3031-4.330) | ***0.005*** | 2.506 (1.282-4.898） | ***0.007*** |
| **Tumor differentiation** |  |  |  |  |
| Well | 1 (/) |  |  |  |
| Moderate | 0.683 (0.311-1.502) | 0.343 |  |  |
| Poor | 1.130 (0.288-4.437) | 0.861 |  |  |
| **Gadd45a expression** |  |  |  |  |
| Negative | 1 (/) |  | 1 (/) |  |
| Positive | 0.518 (0.258-0.941) | ***0.031*** | 0.452 (0.235-0.867) | ***0.017*** |
